# Multiple Sclerosis Incidence Associated with the Soil Lead and Arsenic Concentrations in Taiwan

**DOI:** 10.1371/journal.pone.0065911

**Published:** 2013-06-17

**Authors:** Ching-Piao Tsai, Charles Tzu-Chi Lee

**Affiliations:** 1 Neurology, Neurological Institute, Taipei Veterans General Hospital and National Yang-Ming University, Taipei, Taiwan; 2 Department of Public Health, Kaohsiung Medical University, Kaohsiung, Taiwan; National Institutes of Health, United States of America

## Abstract

**Background:**

Few studies in the world have assessed the incidence of multiple sclerosis (MS) with soil heavy metal concentrations. We explored the association of soil heavy metal factors and the MS incidence in Taiwan.

**Methods:**

There were 1240 new MS cases from the National Health Insurance Research Database and were verified with serious disabling disease certificates, 1997–2008. Soil heavy metal factors records included arsenic, mercury, cadmium, chromium, copper, nickel, lead and zinc in Taiwan from 1986 to 2002. Spatial regression was used to reveal the association of soil heavy metals and age- and gender-standardized incidence ratios for townships by controlling sunlight exposure hours, smoking prevalence and spatial autocorrelation.

**Results:**

The lead (Pb) concentration in the soil positively correlated with the township incidence; on the other hand, the arsenic (As) concentration in soil negatively correlated with the township incidence and when found together controlled each other. The positive correlation of lead (Pb) predominated in males, whereas the negative correlation of arsenic (As) in soil predominated in females.

**Conclusions:**

We conclude that exposure to lead (Pb) in soil positive associated with incidence of MS in Taiwan, especially in males. Exposure to arsenic (As) in soil negative associated with MS in Taiwan, especially in females.

## Introduction

Multiple sclerosis (MS) is an inflammatory demyelization disease of the central nervous system. An increase in the prevalence and incidence of MS has been observed in Europe, Iran and possibly worldwide [1]–[4]. Although its cause is not known, genetic and environmental factors both influence susceptibilities to the disease. However, environmental causes have a predominant impact [5], and family-based genetic epidemiological approaches have found no evidence of non-genetic transmissibility. This indicates that the action of the environment in influencing MS risk is operative at a macro-environmental or population level, and not within families or the microenvironment [6]. Whereas the incidence and prevalence of MS varies geographically [7], little is known about the environmental effects on MS. From the large array of environmental risk factors that have been examined over the past century, infection with Epstein-Barr virus, the ultraviolet light exposure/vitamin D status, and cigarette smoking stand out because of the strength of supporting evidence [8].

Soil heavy metal contamination is a major environmental concern, and the ecological risk associated with heavy metals on human health is increasing. Heavy metals in soil may cause more harm to human health through the food grown in soil. The use of agricultural and residential land poses the highest risk to human health because ingestion is the dominant exposure pathway for heavy metals [9]. Some heavy metals, such as arsenic (As), lead (Pb) and mercury (Hg), are known to play a role in the development of various diseases including cancers [10], [11] and neurological disorders [12]. Relevant to this study, the accumulation of lead (Pb), molybdenum (Mo), arsenic (As) and aluminum (Al) may contribute to the etiology of MS in some cases [13]. We hypothesized that these heavy metals in soil is associated with incidence of MS.

In this study, we firstly to use spatial analysis on population-based data of small geographic regions to investigate the soil heavy metal factors on MS incidence. The results may suggest further research into identifying environmental exposures, and find new approaches for the prevention and perhaps for the treatment of MS.

## Materials and Methods

### National Health Insurance in Taiwan

In 1995, the National Health Insurance (NHI) program, a government-run insurer with a single-payer insurance system, was established in Taiwan. By December 2010, there were 23.074 million individuals enrolled in the program nationwide, with a coverage rate of 99.6%. The registration of all cases of serious disabling diseases (SDD), such as chronic renal failure, myasthenia gravis, cancer, and MS, was required by the bureau of the NHI before certification for SDD could be granted. There were 37,099 registered medical doctors with 553 Neurology specialists in 2008 in Taiwan. In addition, there were 790,621 individuals with SDD certificates in 2008, which constituted 3.4% of the total population.

### Sample

This was a National Health Insurance Database study. The National Health Insurance Research Database (NHIRD), which includes services such as outpatient, ambulatory, hospital inpatient care, and dental, was used in this study. The MS cases were identified according to the International Classification of Disease, ninth revision (ICD-9), code number of 340. The study data were collectedly based on medical claims made by MS patients during 1997 and 2008 from the NHIRD in Taiwan. The MS cases in the SDD list were also included. The study period to include incident MS patients (1997–2008) was depending on the published database of SDD and NHIRD since 1996. The medical claim data in Year 1996 was used to verify our MS patients as new case. The diagnosis of MS was based on Poser’s criteria [14] by in charge clinical neurologist, and their medical records were sent to bureau of the NHI. Neuromyelitis Optica (NMO) was not included in this study. A group of neurology specialists at Taiwan’s bureau of the NHI reviewed the medical records of MS patients using Poser’s criteria [14]. There is no morbidity threshold for a given disease, any patient whole fulfilled Poser’s criteria of MS will be enrolled. As patients with SDD certificates were eligible for exemption from insurance premiums and co-payments, the approval of SDD certificates requires strict evaluation by the bureau of the NHI. In this study, all MS patients were tracked back to their first MS diagnosis date and verifying by linking the encrypted identification numbers with the SDD certificates. Besides, each MS patient had at least one year period to verify as incidence case before they got SDD certificate, there were 1240 new MS cases included in this study, from 1997 to 2008.

### Population Data

The population data was obtained from the year 2000 census in Taiwan by the Directorate-General of Budget, Accounting and Statistics, Executive Yuan, Taiwan. The population of Taiwan’s 358 townships/precincts in the 2000 census was 21.30 million. The resident was 59,490±74,932 (mean±SD) per township, and the average area of townships was 100.9±182.6 (mean±SD) square kilometers. Each township/precinct was treated as a unit in the analysis. Township/precinct of MS patients was defined as their workplace to locate their exposure on soil heavy metal more stable in this study.

### Soil Heavy Metal Data, Sunlight Exposure Hours and the Smoking Prevalence

The most recently national soil survey comparing to our main study period (1997–2008) by Environmental Protection Administration was from 1986 to 2002. In order to investigate the association between the township MS incidence and the soil’s heavy metal factors, the 1986–2002 records of surveys that included arsenic (As), mercury (Hg), cadmium (Cd), chromium (Cr), copper (Cu), nickel (Ni), lead (Pb) and zinc (Zn) for 320 of the total of 358 townships in Taiwan, were obtained from the Environment Protection Administration of the Executive Yuan. Arsenic (As) and mercury (Hg) in the soil were measured with the aqua regia method, cadmium (Cd), chromium (Cr), copper (Cu), nickel (Ni), lead (Pb) and zinc (Zn) was measured with the 0.1 N HCl extraction method. The average of different samples within the same township was served as the representative of the metal concentration in a certain township. For each of the 38 from the total 358 townships without survey data for soil heavy metal, the soil heavy metal factor measurements estimated by the inverse distance weighting (IDW) method [15]. The IDW method is a simplest interpolation method and widely use in the spatial analysis to estimate the smooth measurements across the geographic regions.

Records of annual average hours of sunshine exposure were retrieved from 23 major observation stations of the Central Weather Bureau for the whole of Taiwan. Taiwan is an island 390 kilometers long, and 140 kilometers wide which is located in the northwest Pacific Ocean. Taiwan can be divided into three major geologic provinces: the Central Range, the Western Foothills and the Coastal Range. The Central Range has many mountains and a maximum elevation of almost 4000 meters. The varieties of geographic characteristics in Taiwan let the sunlight exposure with large variation across year. In order to represent the long term sunlight exposure, measurements were calculated during the period from 1981 to 2010.

Because there were only 23 observation stations with long term record of sunlight exposure in Taiwan; and the variation of geographic characteristic between the locations of these weather stations were huge. It did not suitable to estimate each township’s sunshine exposure value by interpolation method. Therefore, each of the 358 townships sunshine exposure hours were directly represented by the record of its nearest observation station in this study. The smoking prevalence of the 358 townships was retrieved from a survey conducted in 2002 by the Department of Health of the Executive Yuan.

### Statistical Analysis

We first included the new 1240 MS cases from the years 1997 to 2008 for the whole of Taiwan based on the National Health Insurance Research database and the serious disabling diseases registry. Secondary, we calculated the indirect age- and gender-standardized incidence rate (ASIR) of MS according to the year 2000 census population of townships/precincts. The expected numbers of MS in each township/precinct were calculated by the weighted average of age-specific MS incidence for the whole of Taiwan and the age distribution of the populations in the townships/precincts. In other words, the expected numbers in the townships/precincts took into account their population and age distribution. The raw values of ASIR were then calculated as a ratio of observed vs. expected numbers of MS cases for the 358 townships/precincts. The analysis values of ASIR were calculated by the inverse distance weighting method from these raw values of ASIR [15]. Controlling for the population and age distribution within each township/precinct, ASIR = 1 indicated the township/precinct incidence was equal to the national incidence average; ASIR>1 indicated the township/precinct incidence was larger than the national incidence average; ASIR<1 indicated the township/precinct incidence was less than the national incidence average.

Spatial regression [16] was used to reveal the association of soil heavy metal factors and MS incidence by controlling sunlight exposure hours, smoking prevalence and spatial autocorrelation. For the spatial data, the correlation between regions needs to be taken into account in the analysis in order to provide valid scientific evidence. Unadjusted, explanatory adjusted (adjusting sunlight exposure hours and smoking prevalence) and the final adjusted model with stepwise selection results is shown in the spatial regression analysis. The stepwise selection to find the most significant factors has been widely used in exploratory analyses, as it has been here. The significance p value was set as 0.05. The model was tested first among the total of cases, then among subgroups according to gender. Paired t test was used to reveal the mean concentration’s difference between pair-wise of heavy metals in soil. Geoda095.i was used to estimate the models and plot the map [17].

## Results

### Characteristic of MS Cases

In the 1,240 new cases of MS patients, there were 290 males and 950 females, and the female-to-male ratio was 3.3. There was a predominantly higher proportion of MS at the diagnosis age of 25–44 years. The mean age of MS diagnosis was 37.9±13.9 (mean±SD). The distribution of MS were 239 (19.27%) in rural and 1001 (80.73%) in urban areas. This distribution was similar to the resident’s distribution, about 2.7 million (13%) residents live in the 145 rural townships, and the other 18.6 million (87%) residents live in the 213 urban townships from the Year 2000 census in Taiwan. For the proxy index of economic status, insurance amount, nearly half of the cases were listed under social support or as a dependent member in the family. Over half the cases were found in the north of Taiwan (Table 1).

**Table 1:** Characteristics of 1,240 MS individuals in Taiwan, 1997–2008.

Characteristic	N	%
**Gender**		
Female	950	76.61
Male	290	23.39
**MS diagnosis age**		
1–24	233	18.79
25–44	616	49.68
45–64	347	27.98
65+	44	3.55
**Residence**		
Rural	239	19.27
Urban	1001	80.73
**Insurance amount**		
Fixed premium and dependent	576	46.45
Less than NTD20,000	313	25.24
NTD20,000 or more	351	28.31
**Geographic region**		
North	670	54.03
Central	217	17.50
South	308	24.84
East	45	3.63

1 US $ = 32.1 NTD in 2008.

### The Soil Heavy Metal Factors

The soil heavy metal factors record from townships in Taiwan shown in Table 2. Although the average arsenic (As) was 5.30 (mg/kg) (SD = 2.62) across Taiwan, because of the geographic diversity of Taiwan, the average concentration in townships widely ranged from 0.01 to 11.42 (mg/kg). Similarly, the average lead (Pb) concentration was 9.14 (mg/kg) (SD = 4.35), with a range of 0.06 to 37.03 (mg/kg). In the study of eight soil heavy metal factors, the free unit variation indicator, coefficient of variation (CV), showed the highest in cadmium (Cd) at 441.03 and the lowest in lead (Pb) at 47.61. The CV of arsenic (As) was 49.38. The high geographic variation of each soil heavy metal concentrations were found in township scale (Table 2). The comparison of mean concentration’s difference between lead (Pb) and the other 7 heavy metals showed that zinc (Zn) was significant higher than lead (Pb) (p<0.001), and copper (Cu) was similar to lead (Pb) (p = 0.274). The other 5 heavy metals’ mean concentrations in soil were all significant to lower than lead (Pb) (p<0.001). In the part of arsenic (As), the mean concentration of zinc (Zn), lead (Pb), and copper (Cu) in soil were significant higher than arsenic (As) (p<0.001). The other 4 heavy metals’ mean concentrations in soil were all significant to lower than arsenic (As) (p<0.001).

**Table 2:** Soil heavy mental statistics of township in Taiwan, 1986–2002.

Soil heavymental (mg/kg)	N	Mean	SD	CV (%)	Minimum	Maximum
As	320	5.30	2.62	49.38	0.01	11.42
Cd	320	0.17	0.74	441.03	0.01	12.91
Cr	320	1.35	2.78	205.98	0.01	19.29
Cu	320	8.69	12.06	138.72	1.12	157.51
Hg	320	0.17	0.13	72.34	0.01	1.19
Ni	320	2.79	2.97	106.16	0.11	29.20
Pb	320	9.14	4.35	47.61	0.06	37.03
Zn	320	13.52	14.72	108.91	1.83	132.51

SD: Standard deviation. CV: Coefficient of variation.

### The Association of Soil Heavy Metal Factors and MS Incidence

**Figure 1:**
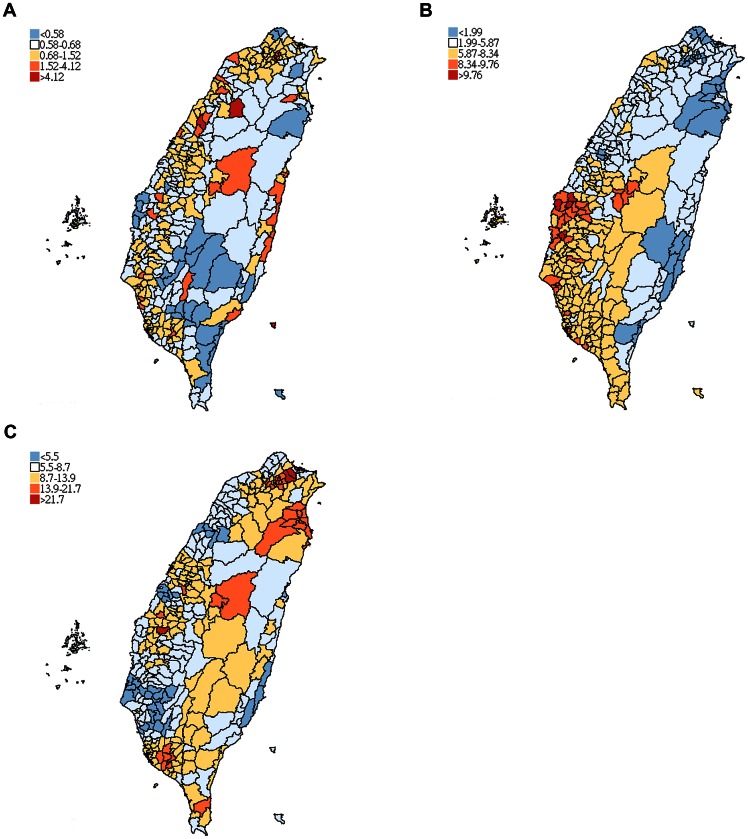
Map of multiple sclerosis age-standardized incidence ratios (ASIR, 1997–2008), soil arsenic (As, 1986–2002) and lead (Pb, 1986–2002) concentrations in Taiwan. (A) ASIR for MS. (B) As (mg/kg). (C) Pb (mg/kg).

In the study of eight soil heavy metal factors, the spatial distribution of MS incidence showed the similar gradient with lead (Pb) but an inverse one for arsenic (As) (Figure 1-ABC). The spatial regression analysis showed the independent predictors of township arsenic (As) and lead (Pb) on total case incidence. Raising one mg/kg in arsenic (As) decreased the township ASIR by 0.044 (p = 0.044) and raising one mg/kg in lead (Pb) increased the township ASIR by 0.025 (p = 0.039) in the final adjusted model. By controlling sunlight exposure and smoking prevalence, the explanatory adjusted analysis indicated lead (Pb) also had a significant positive association (P = 0.034), whereas arsenic (As) had borderline significance (P = 0.052) for the incidence of MS. In the subgroup analysis by gender, however, the greater the lead (Pb) concentration in soil the greater the male incidence; in contrast, the greater the arsenic (As) concentration in soil the lesser the female incidence (Table 3).

**Table 3:** Spatial regression analysis on age-standardized incidence ratios of MS in Taiwan, 1997–2008.

Data	Variable		Unadjusted		Explanatory model		Final adjusted	
			Estimate	P value	Estimate	P value	Estimate	P value
Total cases	Soil heavy mental (mg/kg)	As	−0.0503	0.022	−0.0521	0.052	−0.0444	0.044
		Cd	0.0143	0.858				
		Cr	0.0001	0.996				
		Cu	0.0048	0.322				
		Hg	0.3095	0.494				
		Ni	0.0030	0.881				
		Pb	0.0286	0.020	0.0269	0.034	0.0254	0.039
		Zn	0.0017	0.667				
	Sunlight exposure (100 hours)		−0.0233	0.204	0.0001	0.606		
	Smoking prevalence (%)		0.0002	0.979	0.0002	0.985		
Male cases	Soil heavy mental (mg/kg)	As	−0.0140	0.656				
		Cd	0.0045	0.969				
		Cr	−0.0087	0.777				
		Cu	0.0012	0.867				
		Hg	0.0147	0.982				
		Ni	−0.0185	0.522				
		Pb	0.0362	0.042	0.0349	0.059	0.0362	0.042
		Zn	−0.0008	0.897				
	Sunlight exposure (100 hours)		−0.0233	0.768	0.0001	0.960		
	Smoking prevalence (%)		0.0002	0.254	0.0002	0.345		
Female cases	Soil heavy mental (mg/kg)	As	−0.0798	0.003	−0.0744	0.026	−0.0798	0.003
		Cd	0.0084	0.932				
		Cr	0.0008	0.976				
		Cu	0.0054	0.368				
		Hg	0.3716	0.508				
		Ni	0.0049	0.843				
		Pb	0.0245	0.108				
		Zn	0.0023	0.639				
	Sunlight exposure (100 hours)		−0.0008	0.072	−0.0005	0.867		
	Smoking prevalence (%)		−0.0152	0.296	0.0083	0.470		

Control by spatial autocorrelation.

## Discussion

The primary purpose of this study was to evaluate the association of soil heavy metal factors and MS incidence in Taiwan. The main finding in this study was the high risk spatial clusters of MS incidence were located in the North foothills and East rift valley in Taiwan. The lead (Pb) concentration in soil positively correlated with the township incidence, while the arsenic (As) concentration negatively correlated with the township incidence and when found together controlled each other. The positive correlation of lead (Pb) predominated in males, whereas the negative correlation of arsenic (As) in soil predominated in females.

MS is increasing in incidence mainly in high-income countries. One explanation of this phenomenon may be a higher prevalence of allergic and autoimmune diseases in industrialized countries as a consequence of otherwise beneficial advances in sanitation (hygiene hypothesis) [18]. In this study, the pollutants in soil, especially the lead (Pb), may also play role in the increasing incidence of MS in industrialized countries. A case report study has described a patient with MS treated for neurological symptoms which were thought to be a progression of his disease but which were subsequently found to be caused by lead poisoning; his clinical signs improved with oral chelation therapy [19]. On the other hand, another study revealed that MS cases did not appear to cluster around the lead smelter in Jefferson County, Missouri, USA [20]. In this ecological study, we provided evidences that lead positively correlated with MS incidence. Thus, further investigation is suggested for the effects of lead poisoning on MS patients.

Arsenic was widely used in the past to treat neurological diseases, especially epilepsy and neurosyphilis, and psoriasis [21]. The use of arsenic trioxide in secondary acute promyelocytic leukemia that developed after treatment of MS with mitoxantrone was also described in a recent report [22]. In this study, as arsenic negatively correlated with MS incidence, further research is recommended into the possibility of low dose exposure of arsenic in soil to prevent from MS with the considering of toxic side effects.

Previous research has highlighted the effects of toxic levels of lead on gene regulation in the male central nervous system [23]. In this study, the positive correlation of lead in soil on MS incidence predominated in males. There is a wide variation in the susceptibility to health effects from arsenic, which, in part, may be due to differences in arsenic metabolism. The metabolism of arsenic is higher in men than in women [24]. This may explain why the negative correlation of arsenic predominated in females’ MS incidence with low-dose environmental exposure in this study.

The results in this study need further research in order to identify the mechanism between lead/arsenic and MS. It should be acknowledged that due to the limited number of stations that kept long term sunlight records in Taiwan; a low density of climatic observational stations was a source of limitation in the study. Consequently, this drawback might dilute the controlling of sunlight exposure on the MS incidence in small regions. The workplace exposures to heavy metals may confound with the soil exposures. Migrations can also produce bias because of misclassification of residences. In this ecological study, we did not have the information about people in Taiwan move within, out or into the township. Therefore, township of MS patients was defined as their workplace to locate their exposure on soil heavy metal more stable in this study. Besides, in epidemiological studies, the time of first symptom onset is the traditional starting point for MS. Exposures at or before the time of first symptom onset are most relevant epidemiologically. However, we did not have onset date in our study database. We also absence of some important predictors, such as the symptoms of MS, and leading distinguish of MS patients subtype was not available. Another limitation of this study is that MS is a rare disease in Taiwan, and even though this was a 12 years population-based study, we can only verify 1240 new MS cases. Additionally, MS epidemiology studies focus on childhood and adolescent exposure as being most relevant for developing MS. Unfortunately, the sample size was too small to focus on these age groups’ analyses in this study. We recommend research in a western country with higher MS incidence to perform similar analysis for confirmation.
